# Genetic polymorphisms of pharmacogenomic VIP variants in the Uygur population from northwestern China

**DOI:** 10.1186/s12863-015-0232-x

**Published:** 2015-06-20

**Authors:** Li Wang, Ainiwaer Aikemu, Ayiguli Yibulayin, Shuli Du, Tingting Geng, Bo Wang, Yuan Zhang, Tianbo Jin, Jie Yang

**Affiliations:** Key Laboratory of High Altitude Environment and Genes Related to Diseases of Tibet Autonomous Region, School of Medicine, Xizang Minzu University, Xianyang, Shaanxi 712082 China; Department of Drug Analysis, Faculty of Pharmacy, Xinjiang Medical University, Urumqi, 830054 China; Department of radiotherapy two, The people’s hospital of Xinjiang Uygur Autonomous Region, #91 Tianchi Road, Urumqi, 830001, Xinjiang China; School of Life Sciences, Northwest University, Mailbox 386, #229 North Taibai Road, Xi’an, 710069, Shaanxi China; National Engineering Research Center for Miniaturized Detection Systems, Xi’an, 710069 China

**Keywords:** Pharmacogenomics, genetic polymorphisms, Uygur, VIP variants

## Abstract

**Background:**

Drug response variability observed amongst patients is caused by the interaction of both genetic and non-genetic factors, and frequencies of functional genetic variants are known to vary amongst populations. Pharmacogenomic research has the potential to help with individualized treatments. We have not found any pharmacogenomics information regarding Uygur ethnic group in northwest China. In the present study, we genotyped 85 very important pharmacogenetic (VIP) variants (selected from the PharmGKB database) in the Uygur population and compared our data with other eleven populations from the HapMap data set.

**Results:**

Through statistical analysis, we found that *CYP3A5* rs776746, *VKORC1* rs9934438, and *VKORC1* rs7294 were most different in Uygur compared with most of the eleven populations from the HapMap data set. Compared with East Asia populations, allele A of rs776746 is less frequent and allele A of rs7294 is more frequent in the Uygur population. The analysis of F-statistics (Fst) and population structure shows that the genetic background of Uygur is relatively close to that of MEX.

**Conclusions:**

Our results show significant differences amongst Chinese populations that will help clinicians triage patients for better individualized treatments.

## Background

Reactions to the same drug differ significantly among individuals. Thus, analyzing a drug’s safety and efficacy is complicated, causing difficulties in finding new treatments for major diseases. Inherited differences in individual drug-metabolizing enzymes are typically monogenic traits, and their influence on the pharmacokinetics and pharmacologic effects of medications are determined by the importance of the polymorphic enzymes for the activation or inactivation of drug substrates [1]. Pharmacogenetics and pharmacogenomics deal with possible associations of a single genetic polymorphism or multiple gene profiles and responses to drugs [2]. The goal of pharmacogenetic research is to provide information for a patient with the right medicine at the right dose for optimal treatment outcomes. The majority of pharmacogenomic studies have focused on candidate genes thought to be involved in the pharmacokinetics or mechanism of drug action [3, 4].

Recent studies have shown that certain genes have close relationships with the outcomes of drug therapy and that different genotypes may determine how the patient responds to a drug. These gene variants are called very important pharmacogenetic (VIP) variants [5], and are listed in the Pharmacogenomics Knowledge Base (PharmGKB: http://www.pharmgkb.org). In total, there are 126 VIP variants that occur in 44 different genes and variously code for cytochrome P450 oxidases, drug targets, drug receptors, and drug transporters.

Individual responses to medications vary significantly among different populations, and great progress in understanding the molecular basis of drug actions has been made in the past 50 years. The field of pharmacogenomics seeks to elucidate inherited differences in drug disposition and effects. While we know that different populations and ethnic groups are genetically heterogeneous, we have not found any pharmacogenomics information regarding minority groups, such as the Uygur ethnic group in northwest China.

The Uygur is an ethnic group primarily located in the Xinjiang Uygur Autonomous Region of China. The Uygur is one of China’s largest ethnic groups, with a long history in the region and distinct culture and traditions. They were originally a nomadic Turkish people in north and northwestern China. The Uygur language is a Turkic language very similar to Turkish.

In this study, we aimed to identify the allele frequencies of VIP variants in the Uygur and to determine the difference in allele frequencies between the Uygur and 11 populations from the HapMap data set. The results of this study will extend our understanding of ethnic diversity and pharmacogenomics, and enable medical professionals to use genomic and molecular data to effectively implement personalized medicine in the future.

## Materials and methods

### Study participants

We recruited a random sample of unrelated Uygur adults from the Xinjiang Region of China. The subjects selected were judged to be of good health and had exclusively Uygur ancestry for at least the last three generations. Thus, the subjects were thought to be representative samples of the Uygur population with regard to ancestry and environmental exposures. Blood samples were taken according to the study protocol, which was approved by the Clinical Research Ethics of Northwest University, Tibet University for Nationalities, Xinjiang Medical University, and the people’s hospital of Xinjiang Uygur Autonomous Region. Signed informed consent was also obtained from each participant enrolled in the study. Based on the abovementioned inclusion criteria, 96 randomly-selected, healthy, unrelated Uygur individuals were recruited from the Xinjiang Province.

### Variant selection and genotyping

We selected genetic variants from published polymorphisms associated with VIP variants from the PharmGKB database. We designed assays for the 85 genetically-variant loci in 37 genes that formed the basis for our our analyses. We excluded loci if we could not design an assay. We extracted genomic DNA from peripheral blood obtained from the subjects using the GoldMag-Mini Whole Blood Genomic DNA Purification Kit (GoldMagLtd. Xi’an, China) according to the manufacturer’s protocol. The DNA concentration was measured with a NanoDrop 2000C spectrophotometer (Thermo Scientific, Waltham, MA, USA). The Sequenom MassARRAY Assay Design 3.0 software (San Diego, CA, USA) was used to design multiplexed single nucleotide polymorphism (SNP) MassEXTEND assays [6]. SNP genotyping analysis was performed using the standard protocol recommended by the manufacturer with a Sequenom MassARRAY RS1000. Sequenom Typer 4.0 software was used to manage and analyze the SNP genotyping data as described in a previous report [7].

### HapMap genotype data

The genotype data of individuals from eleven populations were downloaded from the International HapMap Project web site (HapMap_release127) at http://hapmap.ncbi.nlm.nih.gov/biomart/martview/e4f42d4d0acde5ea6c35312381c1e461. The eleven populations included those of (1) African ancestry in Southwest USA (ASW); (2) Utah, USA residents with Northern and Western European ancestry from the CEPH collection (CEU); (3) Han Chinese in Beijing, China (CHB); (4) Chinese in metropolitan Denver, CO, USA (CHD); (5) Gujarati Indians in Houston, Texas, USA (GIH); (6) Japanese in Tokyo, Japan (JPT); (7) Luhya in Webuye, Kenya (LWK); (8) Mexican ancestry in Los Angeles, California, USA (MEX); (9) Maasai in Kinyawa, Kenya (MKK); (10) Toscani in Italy (TSI); and (11) Yoruba in Ibadan, Nigeria (YRI).

### Statistical analyses

We used Microsoft Excel and SPSS 17.0 statistical packages (SPSS, Chicago, IL, USA) to perform Hardy–Weinberg Equilibrium (HWE) analysis and the *χ*^2^ test. The validity of the frequency of each VIP variant in the Uygur data was tested by assessing the departure from HWE using an exact test. We calculated and compared the genotype frequencies of the variants in the Uygur data with those in the eleven populations separately using the *χ*^2^ test. All *p* values obtained in this study were two-sided, and Bonferroni’s adjustment for multiple tests was applied to the level of significance, which was set at *p* < 0.05/(85*11). The purpose of the *χ*^2^ test was to discover sites with significant differences. Afterwards, we obtained the SNP allele frequencies from the ALleleFREquency Database (http://alfred.med.yale.edu), and analyzed the global patterns of genetic variation at specific loci.

### Analysis of population genetic structures

Some studies point out that population genetic structure is central to the study of human origins, DNA forensics, and complex diseases [8]. We believe it is also important for pharmacogenomics. Fst and structure analyses are common in population genetic studies. Because of the insights that F-statistics can provide about the processes of differentiation among populations, over the past 50 years they have become the most widely used descriptive statistics in population and evolutionary genetics [9]. Wright’s F-statistics describe the level of heterozygosity in each level of a hierarchically-subdivided population. More specifically, F-statistics relate the departure from panmixia in the total population and within subpopulations to the total homozygosity. The most commonly reported statistic, Fst, measures the differentiation of a subpopulation relative to the total population, and is directly related to the variance in allele frequency between subpopulations. To further investigate variation at the VIP locus in terms of population structure, we used the model-based clustering method implemented in Structure (http://pritchardlab.stanford.edu/structure.html).

We used the Arlequin ver 3.1 software to calculate the value of Fst to infer the pairwise distance between populations. Pairwise Fst values were calculated on the primary, 84 SNP dataset in Arlequin3.5 [10] using Reynolds’ distance [11] with significance tested using 100 permutations. To further investigate population structure, we used the model-based clustering method implemented in Structure ver. 2.3.1. Fst is directly related to the variance in allele frequency among populations and to the degree of resemblance among individuals within populations. If Fst is small, it means that the allele frequencies within each population are similar; if it is large, it means that the allele frequencies are different.

To analyze the genetic structure, the Bayesian clustering algorithm-based program Structure ver. 2.3.1 was used to assign the samples within a hypothetical K number of populations as proposed by Pritchard et al. [12]. Analyses were performed using the ancestry model with correlated allele frequencies in eleven independent runs from K = 2 to K = 7. The MCMC analyses for each structure analysis (from K = 2 to K = 7) was run for 10,000 steps after an initial burn-in period of 10,000 steps. To assess the most likely number of clusters, we calculated △K following Evanno et al. [13]. When the software ran to completion and results were obtained, we constructed bar charts summarizing the results using drawing software.

## Results

Basic information about the selected VIP loci in Uygur is listed in Table 1. The 85 VIP loci relate to 37 genes that belong to the cytochrome P450 superfamily, the nuclear receptor family, the G-protein coupled receptor family, the alcohol dehydrogenase family, the adrenergic receptors family, the ATP-binding cassette (ABC) transporters superfamily, and the eag family.

**Table 1:** Basic characteristic of selected variants and allele frequencies in the Uygur population

SNP ID	Genes	Family	Phase	Allele A	Allele B	Allele A	Allele B	Amino Acid Translation	Function
rs1801131	MTHFR	methylenetetrahydrofolate reductase family	Phase I	C	A	0.292	0.708	Glu429Ala	Missense
rs1801133	MTHFR	methylenetetrahydrofolate reductase family	Phase I	T	C	0.349	0.651	Ala222Val	Missense
rs890293	CYP2J2	cytochrome P450 superfamily	Phase I	G	T	0.5	0.5	-	5′ Flanking
rs3918290	DPYD	-	PhaseI	G	A	1	0	-	Donor
rs6025	F5	-	Others	G	A	0.979	0.021	Arg534Gln	Missense
rs20417	PTGS2	-	Phase I	G	C	0.99	0.01	-	5′ Flanking
rs689466	PTGS2	-	Phase I	A	G	0.721	0.279	-	5′ Flanking
rs4124874	UGT1A1	UDP-glucuronosyltransferase family	Phase II	C	A	0.474	0.526	-	5′ Flanking
rs10929302	UGT1A1	UDP-glucuronosyltransferase family	Phase II	G	A	0.763	0.237	-	5′ Flanking
rs4148323	UGT1A1	UDP-glucuronosyltransferase family	Phase II	A	G	0.125	0.875	Gly71Arg	Intronic
rs7626962	SCN5A	sodium channel gene family	Others	G	T	1	0	Ser1103Tyr	Missense
rs1805124	SCN5A	sodium channel gene family	Others	G	A	0.193	0.807	Pro1090Leu	Missense
rs6791924	SCN5A	sodium channel gene family	Others	G	A	1	0	Arg34Cys	Missense
rs3814055	NR1I2	nuclear receptor family	Others	C	T	0.641	0.359	-	5′ Flanking
rs2046934	P2RY12	G-protein coupled receptor family	Others	T	C	0.839	0.161	-	Intronic
rs1065776	P2RY1	G-protein coupled receptor family	Others	T	C	0.073	0.927	Ala19Ala	Synonymous
rs701265	P2RY1	G-protein coupled receptor family	Others	G	A	0.219	0.781	Val262Val	Synonymous
rs975833	ADH1A	alcohol dehydrogenase family	Phase I	G	C	0.625	0.375	-	Intronic
rs2066702	ADH1B	alcohol dehydrogenase family	Phase I	C	T	1	0	Arg370Cys	Missense
rs1229984	ADH1B	alcohol dehydrogenase family	Phase I	G	A	0.672	0.328	His48Arg	Missense
rs698	ADH1C	alcohol dehydrogenase family	Phase I	A	G	0.805	0.195	Ile350Val	Missense
rs17244841	HMGCR	-	Phase I	A	T	1	0	-	Intronic
rs3846662	HMGCR	-	Phase I	T	C	0.474	0.526	-	Intronic
rs17238540	HMGCR	-	Phase I	T	G	1	0	-	Intronic
rs1042713	ADRB2	adrenergic receptors family	Phase I	G	A	0.495	0.505	Arg16Gly	Missense
rs1042714	ADRB2	adrenergic receptors family	Phase I	G	C	0.153	0.847	Gln27Glu	Missense
rs1800888	ADRB2	adrenergic receptors family	Phase I	C	T	0.974	0.026	Thr164Ile	Missense
rs1142345	TPMT	methyltransferase superfamily	Phase II	G	A	0.005	0.995	Tyr240Cys	Missense
rs1800460	TPMT	methyltransferase superfamily	Phase II	A	G	0.005	0.995	Ala154Thr	Missense
rs2066853	AHR	-	Others	G	A	0.784	0.216	Arg554Lys	Missense
rs1045642	ABCB1	ATP-binding cassette (ABC) transporters superfamily	Others	T	C	0.574	0.426	Ile1145Ile	Synonymous
rs2032582	ABCB1	ATP-binding cassette (ABC) transporters superfamily	Others	G	T	0.382	0.618	Ser893Ala Ser893Thr	Missense
rs2032582	ABCB1	ATP-binding cassette (ABC) transporters superfamily	Others	G	A	0.806	0.194		
rs2032582	ABCB1	ATP-binding cassette (ABC) transporters superfamily	Others	T	A	0.908	0.092		
rs1128503	ABCB1	ATP-binding cassette (ABC) transporters superfamily	Others	T	C	0.667	0.333	Gly412Gly	Synonymous
rs10264272	CYP3A5	cytochrome P450 superfamily	Phase I	C	T	1	0	Lys208Lys	Not Available
rs776746	CYP3A5	cytochrome P450 superfamily	Phase I	G	A	0.984	0.016	-	Acceptor
rs4986913	CYP3A4	cytochrome P450 superfamily	Phase I	C	T	1	0	Pro467Ser	Missense
rs4986910	CYP3A4	cytochrome P450 superfamily	Phase I	T	C	1	0	Met445Thr	Missense
rs4986909	CYP3A4	cytochrome P450 superfamily	Phase I	C	T	1	0	Pro416Leu	Missense
rs12721634	CYP3A4	cytochrome P450 superfamily	Phase I	T	C	1	0	Leu15Pro	Missense
rs2740574	CYP3A4	cytochrome P450 superfamily	Phase I	A	G	0.984	0.016	-	5′ Flanking
rs3815459	KCNH2	eag family	Others	A	G	0.564	0.436	-	Intronic
rs36210421	KCNH2	eag family	Others	G	T	1	0	Arg707Leu	Missense
rs12720441	KCNH2	eag family	Others	C	T	1	0	Arg444Trp	Missense
rs3807375	KCNH2	eag family	Others	A	G	0.521	0.479	-	Intronic
rs4986893	CYP2C19	cytochrome P450 superfamily	Phase I	G	A	0.974	0.026	Trp212null	Stop Codon
rs4244285	CYP2C19	cytochrome P450 superfamily	Phase I	G	A	0.828	0.172	Pro227Pro	Synonymous
rs1799853	CYP2C9	cytochrome P450 superfamily	Phase I	C	T	1	0	Arg144Cys	Missense
rs1801252	ADRB1	adrenergic receptors family	Phase I	G	A	0.167	0.833	Ser49Gly	Missense
rs1801253	ADRB1	adrenergic receptors family	Phase I	C	G	0.813	0.188	Gly389Arg	Missense
rs5219	KCNJ11	inward-rectifier potassium channel family	Others	C	T	0.688	0.312	Lys23Glu	Intronic
rs1695	GSTP1	glutathione S-transferase family	Phase II	A	G	0.683	0.317	Ile105Val	Missense
rs1138272	GSTP1	glutathione S-transferase family	Phase II	T	C	0.058	0.942	Ala114Val	Missense
rs1800497	ANKK1	Ser/Thr protein kinase family	Phase I	T	C	0.253	0.747	Glu713Lys	Missense
rs6277	DRD2	G-protein coupled receptor family	Others	C	T	0.656	0.344	Pro290Pro	Synonymous
rs4149056	SLCO1B1	solute carrier family	Others	T	C	0.889	0.111	Val174Ala	Missense
rs7975232	VDR	nuclear receptor family	Others	C	A	0.615	0.385	-	Intronic
rs1544410	VDR	nuclear receptor family	Others	G	A	0.74	0.26	-	Intronic
rs2239185	VDR	nuclear receptor family	Others	T	C	0.395	0.605	-	Intronic
rs1540339	VDR	nuclear receptor family	Others	G	A	0.5	0.5	-	Intronic
rs2239179	VDR	nuclear receptor family	Others	A	G	0.62	0.38	-	Intronic
rs3782905	VDR	nuclear receptor family	Others	C	G	0.742	0.258	-	Intronic
rs2228570	VDR	nuclear receptor family	Others	T	C	0.316	0.684	Met51Arg,Met51Lys,Met51Thr	Missense
rs10735810	VDR	nuclear receptor family	Others	C	T	0.688	0.313	-	-
rs11568820	VDR	nuclear receptor family	Others	G	A	0.658	0.342	-	Not Available
rs1801030	SULT1A1	sulfotransferase family	Phase II	A	G	1	0	Val223Met	Not Available
rs3760091	SULT1A1	sulfotransferase family	Phase II	C	G	0.659	0.341	-	5′ Flanking
rs7294	VKORC1	-	Phase I	G	A	0.695	0.305	-	3′ UTR
rs9934438	VKORC1	-	Phase I	G	A	0.427	0.573	-	Intronic
rs28399454	CYP2A6	cytochrome P450 superfamily	Phase I	G	A	1	0	Val365Met	Missense
rs28399444	CYP2A6	cytochrome P450 superfamily	Phase I	AA	-	1	0	Glu197Ser,Glu197Arg	Frameshift
rs1801272	CYP2A6	cytochrome P450 superfamily	Phase I	T	A	1	0	Leu160His	Missense
rs28399433	CYP2A6	cytochrome P450 superfamily	Phase I	G	T	0.13	0.87	-	5′ Flanking
rs3745274	CYP2B6	cytochrome P450 superfamily	Phase I	G	T	0.792	0.208	Gln172His	Missense
rs28399499	CYP2B6	cytochrome P450 superfamily	Phase I	T	C	1	0	Ile328Thr	Missense
rs3211371	CYP2B6	cytochrome P450 superfamily	Phase I	C	T	0.495	0.505	Arg487Cys	Missense
rs12659	SLC19A1	solute carrier family	Others	C	T	0.589	0.411	Pro192Pro	Synonymous
rs1051266	SLC19A1	solute carrier family	Others	G	A	0.579	0.421	His27Arg	Missense
rs1131596	SLC19A1	solute carrier family	Others	T	C	0.872	0.128	-	5′ UTR
rs4680	COMT	-	Phase II	A	G	0.432	0.568	Val158Met	5′ Flanking
rs59421388	CYP2D6	cytochrome P450 superfamily	Phase I	C	T	1	0	Val287Met	Missense
rs28371725	CYP2D6	cytochrome P450 superfamily	Phase I	G	A	0.896	0.104	-	Intronic
rs16947	CYP2D6	cytochrome P450 superfamily	Phase I	G	A	0.726	0.274	-	Not Available
rs61736512	CYP2D6	cytochrome P450 superfamily	Phase I	C	A/G/T	1	0	Val136Met	Intronic
rs28371706	CYP2D6	cytochrome P450 superfamily	Phase I	C	T	1	0	Thr107Ile	Missense
rs5030656	CYP2D6	cytochrome P450 superfamily	Phase I	AAG	-	1	0	-	Non-synonymous

Using the *χ*^2^ test with the Bonferroni correction for multiple hypotheses and multiple comparisons, we found 0, 1, 3, 5, 7, 9, 10, 13, 16, 17, and 25 different loci in the frequency distributions when the Uygur population was compared to the TSI, MEX, GIH, CHD, CEU, CHB, ASW, JPT, MKK, LWK, and YRI populations, respectively. Three loci (rs776746, rs9934438, and rs7294) located in the *CYP3A5* and *VKORC1* genes were different in the Uygur population when compared with most of the populations (Tables 2 and 3).

**Table 2:** Significant variants in Uygur compared to the 11 populations, as determined by Chi-square test

SNP ID	Genes	Chi-square test *p* value										
		CHB	JPT	CEU	YRI	ASW	CHD	GIF	LWK	MEX	MKK	TSI
rs1801131	*MTHFR*	2.64E-01	5.50E-02	5.61E-01	4.64E-05	1.28E-01	1.28E-01	1.23E-01	5.13E-02	3.21E-01	6.99E-01	4.68E-01
rs1801133	*MTHFR*	5.56E-02	8.61E-01	6.49E-01	5.64E-09	6.93E-06	9.87E-01	6.81E-04	4.89E-08	4.45E-01	4.97E-11	7.77E-02
rs6025	*F5*	-	-	5.47E-01	-	-	-	-	-	-	-	-
rs20417	*PTGS2*	2.27E-30	3.82E-30	1.42E-30	3.59E-25	-	-	-	-	-	-	-
rs689466	*PTGS2*	1.58E-04	9.82E-03	1.79E-02	8.07E-06	2.72E-03	3.17E-03	1.18E-02	4.71E-09	6.02E-01	6.96E-13	3.42E-02
rs4124874	*UGT1A1*	5.43E-04	2.54E-02	8.95E-01	1.45E-18	6.07E-06	2.73E-02	1.58E-02	2.31E-14	6.95E-01	8.73E-14	5.94E-01
rs10929302	*UGT1A1*	1.27E-02	1.55E-02	7.21E-01	2.68E-02	-	-	-	-	-	-	-
rs4148323	*UGT1A1*	1.00E-02	8.23E-01	3.34E-04	3.34E-04	-	4.91E-01	1.52E-03	-	4.21E-02	-	-
rs7626962	*SCN5A*	-	-	-	1.61E-03	-	-	-	-	-	-	-
rs1805124	*SCN5A*	5.41E-02	1.67E-01	7.66E-01	3.09E-02	3.68E-01	7.42E-03	9.77E-01	1.04E-01	5.81E-01	1.01E-03	4.23E-01
rs3814055	*NR1I2*	2.86E-01	1.37E-01	8.69E-01	1.21E-01	7.00E-01	7.20E-02	4.08E-01	2.75E-01	2.51E-01	8.66E-04	8.24E-01
rs2046934	*P2RY12*	6.84E-01	6.10E-01	2.60E-01	2.50E-01	-	-	-	-	-	-	-
rs701265	*P2RY1*	2.09E-01	5.56E-01	6.25E-01	2.75E-23	7.57E-11	4.26E-01	2.11E-01	4.41E-21	8.25E-01	1.87E-23	4.24E-01
rs975833	*ADH1A*	7.76E-11	3.63E-09	2.56E-01	2.56E-01	-	-	-	-	-	-	-
rs2066702	*ADH1B*	-	-	-	1.70E-14	2.43E-10	-	-	7.05E-07	-	-	-
rs1229984	*ADH1B*	4.84E-10	6.69E-09	1.28E-11	1.79E-11	-	-	-	-	-	-	-
rs698	*ADH1C*	2.29E-04	4.26E-04	5.04E-08	1.35E-04	5.01E-02	3.58E-03	2.41E-01	3.71E-01	5.69E-01	4.18E-01	2.40E-02
rs3846662	*HMGCR*	7.31E-01	9.72E-01	6.50E-02	1.61E-21	1.18E-08	6.07E-01	3.68E-02	8.13E-20	2.60E-02	2.51E-12	1.88E-01
rs1042713	*ADRB2*	5.37E-01	2.62E-01	7.49E-03	2.31E-01	4.76E-01	3.59E-01	1.81E-01	5.87E-01	6.38E-01	6.13E-01	2.35E-03
rs1042714	*ADRB2*	6.84E-01	7.77E-02	5.86E-08	3.04E-01	-	-	-	-	-	-	-
rs1142345	*TPMT*	-	-	-	6.38E-02	-	-	-	5.08E-05	4.66E-03	7.52E-52	-
rs2066853	*AHR*	6.40E-04	9.34E-06	5.30E-03	1.34E-05	2.98E-03	2.09E-03	2.84E-02	5.09E-07	1.08E-01	1.26E-03	3.22E-03
rs1045642	*ABCB1*	8.23E-03	3.13E-02	3.10E-01	3.16E-18	7.87E-08	1.84E-04	9.12E-01	-	1.07E-01	4.28E-17	1.33E-01
rs2032582	*ABCB1*	8.02E-01	3.09E-01	9.05E-03	-	1.06E-14	3.95E-02	-	-	-	-	-
rs2032582	*ABCB1*	-	-	-	-	1.49E-01	1.77E-04	-	-	-	-	-
rs2032582	*ABCB1*	-	-	-	-	1.23E-16	1.51E-10	-	-	-	-	-
rs1128503	*ABCB1*	7.10E-01	2.93E-01	1.73E-05	1.63E-22	4.13E-12	7.84E-01	2.67E-01	1.51E-20	2.52E-03	5.63E-23	6.23E-05
rs10264272	*CYP3A5*	-	-	-	1.76E-08	-	-	-	3.72E-12	-	1.61E-07	-
rs776746	*CYP3A5*	4.82E-13	1.37E-12	5.51E-02	1.56E-43	9.11E-27	2.09E-10	1.04E-10	9.92E-38	3.17E-11	2.71E-28	1.52E-02
rs3815459	*KCNH2*	4.49E-02	6.90E-04	-	2.69E-03	-	-	-	-	-	-	-
rs3807375	*KCNH2*	9.10E-04	8.15E-08	8.52E-03	2.75E-07	1.82E-02	5.77E-04	1.94E-02	3.81E-07	6.07E-01	1.36E-05	2.76E-03
rs4244285	*CYP2C19*	7.60E-03	7.79E-02	7.63E-01	8.20E-01	-	-	-	-	-	-	-
rs1801252	*ADRB1*	3.99E-04	4.69E-04	-	1.77E-04	-	-	-	-	-	-	-
rs1801253	*ADRB1*	4.01E-01	5.97E-01	4.11E-02	1.39E-04	-	-	-	-	-	-	-
rs1695	*GSTP1*	1.97E-02	5.30E-06	4.87E-02	2.14E-01	5.59E-02	3.14E-02	5.37E-01	2.61E-04	1.46E-03	5.49E-01	5.90E-01
rs1138272	*GSTP1*	-	-	-	-	-	-	-	-	3.82E-01	-	-
rs1800497	*ANKK1*	5.75E-03	7.02E-03	4.03E-01	2.26E-03	3.61E-02	1.02E-03	9.04E-01	4.19E-02	7.81E-03	3.21E-02	5.35E-01
rs6277	*DRD2*	7.51E-07	9.21E-07	2.73E-03	1.15E-09	-	-	-	-	-	-	-
rs4149056	*SLCO1B1*	3.90E-01	2.55E-01	3.73E-01	-	3.47E-02	3.12E-01	-	-	-	4.92E-01	1.43E-02
rs7975232	*VDR*	3.64E-01	5.13E-01	2.26E-03	7.99E-06	3.56E-04	2.20E-01	7.13E-03	5.31E-09	5.87E-02	8.30E-09	7.35E-04
rs1544410	*VDR*	7.55E-08	2.36E-03	6.90E-04	8.50E-01	2.39E-01	5.95E-08	3.45E-03	9.28E-01	9.17E-01	3.44E-02	6.49E-03
rs2239185	*VDR*	2.76E-01	3.57E-01	-	4.96E-02	-	-	-	-	-	-	-
rs1540339	*VDR*	4.18E-04	7.39E-05	2.87E-02	2.34E-08	2.07E-04	2.63E-05	1.43E-02	3.95E-11	2.23E-01	7.15E-11	2.40E-02
rs2239179	*VDR*	1.49E-02	4.20E-03	3.05E-02	1.57E-01	1.22E-01	4.13E-03	7.35E-02	7.84E-01	2.95E-01	4.02E-01	7.32E-01
rs3782905	*VDR*	3.53E-13	2.82E-17	1.09E-10	2.88E-14	-	-	-	-	-	-	-
rs10735810	*VDR*	1.61E-01	1.87E-01	9.34E-02	1.81E-02	4.22E-02	4.66E-03	5.86E-01	1.58E-03	2.77E-03	1.90E-02	2.90E-01
rs11568820	*VDR*	1.28E-01	8.00E-02	6.18E-03	1.16E-31	5.41E-08	8.53E-01	3.03E-01	3.47E-19	3.45E-02	6.79E-17	7.59E-02
rs7294	*VKORC1*	4.64E-08	2.30E-05	3.77E-01	5.06E-05	2.06E-03	7.51E-07	1.38E-12	1.46E-02	7.81E-01	1.90E-04	3.15E-01
rs9934438	*VKORC1*	3.05E-12	2.10E-09	4.69E-03	2.89E-26	1.19E-11	2.46E-11	4.83E-11	9.77E-19	1.61E-01	3.19E-16	9.26E-02
rs1801272	*CYP2A6*	-	1.08E-30	3.63E-34	-	-	-	-	-	-	-	-
rs3745274	*CYP2B6*	3.21E-01	2.23E-01	1.95E-01	2.27E-05	2.73E-01	1.80E-01	6.90E-05	3.31E-02	2.34E-01	4.15E-04	1.30E-01
rs28399499	*CYP2B6*	-	-	-	3.33E-06	4.73E-04	-	-	-	-	1.80E-01	-
rs1051266	*SLC19A1*	8.64E-03	2.37E-03	4.08E-01	3.20E-09	2.10E-01	2.71E-01	4.03E-01	3.44E-10	3.83E-02	1.97E-14	4.33E-02
rs4680	*COMT*	4.53E-02	8.67E-03	5.32E-01	1.75E-02	1.64E-02	2.29E-03	9.75E-01	5.38E-02	6.03E-01	3.36E-03	3.41E-01

*p* <0.05 indicates statistical significance

**Table 3:** Number of variants significantly different from the 11 populations and corresponding gene families after correction for multiple tests

Gene Family	Significant Variants (N)										
	TSI	MEX	GIH	CHD	CEU	CHB	ASW	JPT	MKK	LWK	YRI
methylenetetrahydrofolate reductase family	0	0	0	0	0	0	0	0	2	2	2
cytochrome P450 superfamily	0	1	1	2	1	1	1	2	2	2	4
UDP-glucuronosyltransferase family	0	0	0	0	0	0	1	0	1	1	1
sodium channel gene family	0	0	0	0	0	0	0	0	0	0	0
nuclear receptor family	0	0	0	2	1	2	1	1	3	3	4
G-protein coupled receptor family	0	0	0	0	0	0	1	0	1	1	0
alcohol dehydrogenase family	0	0	0	0	2	2	1	2	0	1	2
adrenergic receptors family	0	0	0	0	1	0	0	0	0	0	0
methyltransferase superfamily	0	0	0	0	0	0	1	0	0	0	0
ATP-binding cassette (ABC) transporters superfamily	0	0	0	0	1	0	2	0	2	1	2
eag family	0	0	0	0	0	0	0	1	1	1	1
inward-rectifier potassium channel family	0	0	0	0	0	0	0	0	0	0	0
glutathione S-transferase family	0	0	0	0	0	0	0	1	0	0	0
Ser/Thr protein kinase family	0	0	0	0	0	0	0	0	0	0	0
G-protein coupled receptor family	0	0	0	0	0	1	0	1	0	0	2
solute carrier family	0	0	0	0	0	0	0	0	0	1	1
sulfotransferase family	0	0	0	0	0	0	0	0	1	0	0
-	0	0	2	1	1	3	2	4	3	4	6
Sum	0	1	3	5	7	9	10	12	16	17	25

For a global analysis, we combined our new data with previously published data, for a total of 66 population samples at rs776746 and rs7294. From Table 4 it can clearly be seen that the frequencies of the A allele of rs776746 were higher in Africa than in Asia and East Asia, but lower in Europe. For the East Asia data, frequencies ranged from 5 % to 50 %, and the frequencies were high in the She and Tujia population and lower in the Uygur and Tu populations. The frequencies of the A allele of rs7294 in East Asia ranged from 1 % to 35 %, and the frequency in the Uygur population was higher than in the other populations from East Asia.

**Table 4:** Allele frequencies of rs776746 and rs7294 in populations from different regions of the world

Geographic Region	Population	CYP3A5rs776746		VKORC1rs7294	
		Allele A frequency	Allele G frequency	Allele A frequency	Allele G frequency
Africa	Bantu speakers	0.81	0.19	0.38	0.63
	Bantu speakers	0.83	0.17	0.67	0.33
	San	0.92	0.08	0.33	0.67
	Biaka	0.94	0.06	0.81	0.19
	Mbuti	0.93	0.07	0.83	0.17
	Yoruba	0.94	0.06	0.50	0.50
	Mandenka	0.69	0.31	0.56	0.44
	Mozabite	0.15	0.85	0.27	0.73
Asia	Bedouin	0.15	0.85	0.30	0.70
	Druze	0.09	0.91	0.21	0.79
	Palestinian	0.18	0.82	0.28	0.72
	Burusho	0.22	0.78	0.62	0.38
	Kalash	0.24	0.76	0.30	0.70
	Pashtun	0.13	0.87	0.70	0.30
	Mongolian	0.35	0.65	0.15	0.85
	Balochi	0.20	0.80	0.52	0.48
	Balochi	0.14	0.86	0.50	0.50
	Brahui	0.12	0.88	0.48	0.52
	Hazara	0.25	0.75	0.21	0.79
	Sindhi	0.22	0.78	0.52	0.48
	Oroqen	0.15	0.85	0.00	1.00
East Asia	Dai	0.45	0.55	0.20	0.80
	Daur	0.11	0.89	0.06	0.94
	Han	0.26	0.74	0.01	0.99
	Hezhe	0.17	0.83	0.17	0.83
	Japanese	0.23	0.77	0.09	0.91
	Koreans	0.19	0.82	0.05	0.95
	Lahu	0.30	0.70	0.15	0.85
	Miao	0.35	0.65	0.20	0.80
	Naxi	0.22	0.78	0.11	0.89
	She	0.45	0.55	0.25	0.75
	Tu	0.10	0.90	0.10	0.90
	Tujia	0.50	0.50	0.05	0.95
	Uyghur	0.05	0.95	0.35	0.65
	Xibe	0.22	0.22	0.17	0.83
	Yi	0.20	0.80	0.15	0.85
	Cambodians, Khmer	0.27	0.73	0.14	0.86
Europe	Adygei	0.12	0.88	0.15	0.85
	Basque	0.04	0.96	0.28	0.72
	Estonian	0.08	0.92	0.41	0.59
	French	0.09	0.91	0.28	0.72
	Italians	0.06	0.94	0.50	0.50
	Italians	0.19	0.81	0.31	0.69
	Orcadian	0.16	0.84	0.38	0.63
	Russians	0.06	0.94	0.36	0.64
	Sardinian	0.04	0.96	0.32	0.68
North America	Pima, Mexico	0.54	0.46	0.48	0.52
	Maya, Yucatan	0.30	0.70	0.64	0.36
Oceania	Papuan New Guinean	0.21	0.79	0.74	0.24
	Melanesian, Nasioi	0.18	0.82	0.66	0.34
Siberia	Yakut	0.10	0.90	0.06	0.94
South America	Amerindians	0.15	0.85	0.31	0.69
	Karitiana	0.23	0.77	0.79	0.21
	Surui	0.17	0.83	0.40	0.60

Pairwise Fst values were calculated for all population comparisons across loci. As shown in Table 5, we found that pairwise Fst values for comparisons of the Uygur population with the other 11 populations ranged from 0.49686 to 0.581. Fst is directly related to the variance in allele frequency among populations and to the degree of resemblance among individuals within populations. If Fst is small, it means that the allele frequencies within each population are similar; if it is large, it means that the allele frequencies are different. The value of Fst for the Uygur and MEX populations was the smallest. We therefore conclude that the allele frequencies of the Uygur and MEX are similar. We speculate that the genetic backgrounds of the Uygur and MEX populations are similar.

**Table 5:** Fst values between population pairs

	Uygur	ASW	CEU	CHB	CHD	GIH	JPT	LWK	MEX	MKK	TSI	YRI
Uygur	0											
ASW	0.53235	0										
CEU	0.50418	0.15651	0									
CHB	0.52377	0.20398	0.13482	0								
CHD	0.52714	0.20593	0.12811	−0.0009	0							
GIH	0.50346	0.09725	0.03652	0.16088	0.15637	0						
JPT	0.52382	0.18675	0.12683	0.00348	0.00521	0.14951	0					
LWK	0.56694	0.02014	0.23624	0.28267	0.28819	0.17427	0.26257	0				
MEX	0.49686	0.12632	0.02647	0.08544	0.0786	0.05464	0.08481	0.21135	0			
MKK	0.54064	0.01817	0.15704	0.22475	0.22848	0.10714	0.20085	0.02468	0.15325	0		
TSI	0.49987	0.15367	0.00183	0.11417	0.11244	0.04155	0.10694	0.23517	0.0262	0.15761	0	
YRI	0.581	0.01805	0.24612	0.28525	0.29191	0.17483	0.26311	0.00481	0.22153	0.02523	0.24647	0

**Fig. 1:**
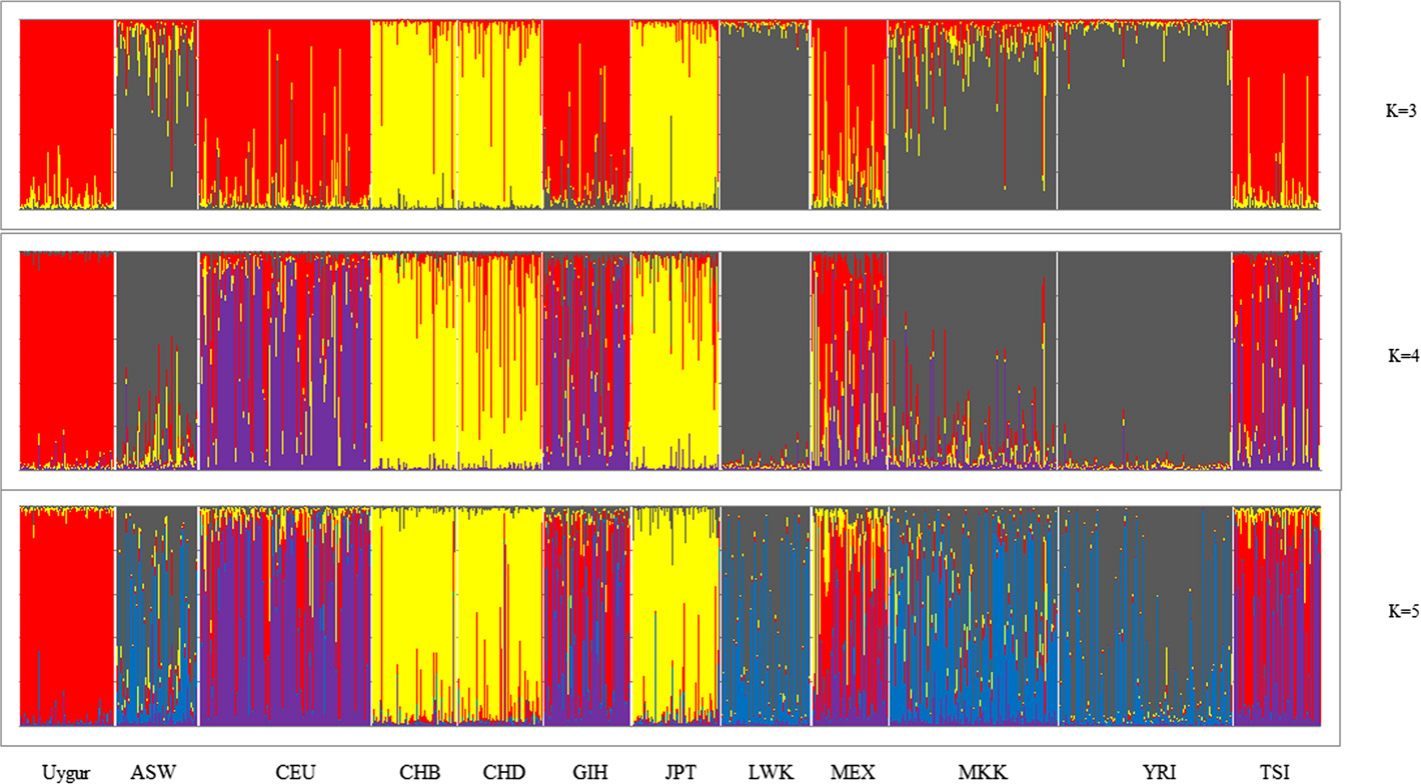
Bayesian clustering of genotypic samples from 12 populations. Each vertical bar denotes an individual, whilst colors denote inferred clusters. Note that colors are not universal between k = 3 and 5

We used a model-based clustering approach, as implemented in Structure, to infer population structure among the 12 populations. Different values ranging from 2 to 7 were assumed for K in Structure calculations. K = 3, 4, 5 were selected, based on the Estimated Ln Prob of Data and other recommendations of the Structure software manual. As shown in Fig. 1, when the K value was equal to 3, individuals were independently assigned to three affinity groups (subpopulations 1: Uygur, CEU, GIH, MEX, TSI; subpopulations 2: ASW, LWK, MKK, YRI; subpopulations 3: CHB, CHD, JPT) using the relative majority of likelihood to assign individuals to subpopulations. We tested additional values of K and obtained results suggesting that the genetic backgrounds of the Uygur and MEX populations are simila.

## Discussion

The genotype frequencies of VIP variants differs among human populations. In this study, we genotyped the variants related to drug response in the Uygur ethnic group and compared the genotype frequencies with those in eleven populations. From the *χ*^2^ test, we found clear evidence that the allele characteristics of the *CYP3A5* rs776746 and *VKORC1* (rs9934438 and rs7294) variants in the Uygur population are quite different from that in other ethnic groups. We also found that the genetic backgrounds of the Uygur and MEX populations are similar, via Fst calculations and analysis of population structure.

*CYP3A5*, localized on chromosome 7q21-q22.1, encodes one of the CYP3A subfamily of enzymes [14]. The most common nonfunctional variant of *CYP3A5* is designated as CYP3A5*3. CYP3A5*3 status is determined by the derived allele at rs776746, a change from A to G located in intron 3. This change creates a cryptic splice site that results in altered mRNA splicing, which may alter the reading frame and result in a premature termination codon and hence a nonfunctional protein [14, 15].

Individuals with CYP3A5*1/*1 and *1/*3 expresser genotypes metabolize some CYP3A substrates more rapidly than CYP3A5*3/*3 nonexpressers. One such substrate is tacrolimus, which is used to prevent post-transplantation organ rejection. CYP3A5*1 carriers have a higher rate of tacrolimus clearance than those with the other genotypes, with *1/*1 individuals having a higher clearance than *1/*3 individuals, who have higher clearance than *3/*3 individuals [16]. In ideal situations, the target tacrolimus concentration must be high enough to prevent transplant rejection [17, 18], but low enough to minimize toxicity [19]. Tacrolimus trough concentrations are routinely monitored after transplantation, and the dose is appropriately adjusted.

Carbamazepine (CBZ), a first-line antiepileptic drug, has been widely prescribed for the treatment of partial and generalized tonic-clonic seizures. It has been reported that CYP3A5*3 is associated with CBZ pharmacokinetics in Japanese [20], Korean [21], and Chinese [22] epileptic patients, and that *CYP3A5* expressers are more likely to require higher CBZ maintenance doses than nonexpressers (GA + AA vs. GG). The *CYP3A5* genotype may also have dose-dependent effects on ABT-773 plasma levels. *CYP3A5* expressers have a higher rate of ifosfamide N-demethylation in the liver and kidney and of cyclosporine A metabolism in the kidney [15].

CYP3A5*3 is the most frequent and well-studied variant allele of *CYP3A5*. Its frequency varies widely across human populations. In white populations, the estimated allele G frequency of CYP3A5*3 is 0.82–0.95, in African American is 0.33, in Japanese is 0.85, in Chinese is 0.65, in Mexicans is 0.75, in Pacific Islanders is 0.65, and in Southwest American Indians is 0.4 [15]. In our study, the frequency of allele G is higher than in other population from China. This suggests that ancestry should be considered when determining dosages for different patients.

The *VKORC1* (vitamin K epoxide reductase complex, subunit 1) gene, which encodes vitamin K epoxide reductase complex subunit 1, located on chromosome 16, includes three exons [23]. The 1173C > T (rs9934438) transition in intron 1 and the 3730G > A (rs7294) transition in the 3ʹ untranslated region (UTR), are two common polymorphisms [24].

Several authors have shown that acenocoumarol dose is also influenced by *VKORC1* genotype. Reitsma et al. showed in 2005 that Dutch patients carrying one or two variant alleles for the 1173 polymorphism required a 28 % and 47 % lower dose, respectively, when compared with wild types [25]. In Greek acenocoumarol users, heterozygous carriers of a variant allele required a 19 % lower dose and homozygous carriers a 63 % lower dose [26]. Similar percentages were found in a German and Austrian population (25 % and 52 %) [27], in a Serbian population (27 % and 62 %) [28], and amongst Lebanese acenocoumarol users (34 % and 50 %) [29]. Reitsma et al. also investigated the influence of *VKORC1* polymorphism on phenprocoumon dose requirements. Patients with a CT genotype at position 1173 had a 10 % lower dose and patients with a TT genotype a 52 % lower dose than wild types (CC) [25]. This effect was also seen in several German and Austrian studies. The dose in phenprocoumon users with one variant *VKORC1* allele was 19–31 % lower than in wild type users, and 43–51 % lower in users with two variant alleles [27].

Warfarin is a commonly prescribed oral anticoagulant, used to prevent thromboembolic diseases in patients with deep vein thrombosis, atrial fibrillation, recurrent stroke, or heart valve prosthesis [30]. Some studies have suggested that carriers of the 1173TT genotype require a dose of warfarin significantly lower than that of carriers with the CC or CT genotypes [24]. On the other hand, the 3730G > A polymorphism was associated with differences in the average dose of warfarin prescribed, with patients carrying the GG genotype being prescribed a significantly lower average daily dose of warfarin [24, 31].

In summary, *VKORC1* polymorphisms can significantly alter warfarin pharmacodynamics and maintenance dose requirements. Patients with the 1173T (rs9934438) allele require a lower warfarin dose compared with 35 mg/week for the wild-type carriers [32]. Patients with 3730A (rs7294) need a higher warfarin dose [32, 33]. In our study, the frequency of carriers of the allele T of rs9934438 and allele G of rs7294 are lower than in other Asian populations, and higher than in European and YRI populations, which suggests that the optimal dosage of warfarin should be decided based on the specific genotype in individual Uygur patients.

## Conclusion

The genotype frequencies of VIP variants affect a populations’ response to drugs to a great extent. Determination of the genotype distribution and frequencies of VIP variants in a population is necessary to provide a theoretical basis for safer drug administration and an improved curative effect. Our results complement the currently available data on the Uygur ethnic group in the pharmacogenomics database, and furthermore, provide a basis for safer and more effective drug administration in the Uygur. However, our sample size of Uygur is relatively small, and further investigation in a larger cohort of Uygur is necessary to ascertain the generalizability and extrapolation of our results to these and other conditions in the Uygur population.

## Abbreviations

VIP variants: very important pharmacogenetic variants
SNP: single nucleotide polymorphism
ASW: African ancestry in Southwest USA
CEU: Utah residents with Northern and Western European ancestry from the CEPH collection
CHB: Han Chinese in Beijing, China
CHD: Chinese in Metropolitan Denver, Colorado
GIH: Gujarati Indians in Houston, Texas
JPT: Japanese in Tokyo, Japan
LWK: Luhya in Webuye, Kenya
MEX: Mexican ancestry in Los Angeles, California
MKK: Maasai in Kinyawa, Kenya
TSI: Toscans in Italy
YRI: Yoruba in Ibadan, Nigeria (West Africa)
